# Thermally and Electrically Conductive Nanopapers from Reduced Graphene Oxide: Effect of Nanoflakes Thermal Annealing on the Film Structure and Properties

**DOI:** 10.3390/nano7120428

**Published:** 2017-12-05

**Authors:** M. Mar Bernal, Mauro Tortello, Samuele Colonna, Guido Saracco, Alberto Fina

**Affiliations:** 1 Dipartimento di Scienza Applicata e Tecnologia, Politecnico di Torino, 15121 Alessandria, Italy; maria.bernal@polito.it (M.M.B.); samuele.colonna@polito.it (S.C.); 2 Dipartimento di Scienza Applicata e Tecnologia, Politecnico di Torino, 10129 Torino, Italy; mauro.tortello@polito.it (M.T.); guido.saracco@polito.it (G.S.)

**Keywords:** reduced graphene oxide, nanopapers, thermal annealing, in-plane thermal conductivity, electrical conductivity

## Abstract

In this study, we report a novel strategy to prepare graphene nanopapers from direct vacuum filtration. Instead of the conventional method, i.e., thermal annealing nanopapers at extremely high temperatures prepared from graphene oxide (GO) or partially reduced GO, we fabricate our graphene nanopapers directly from suspensions of fully reduced graphene oxide (RGO), obtained after RGO and thermal annealing at 1700 °C in vacuum. By using this approach, we studied the effect of thermal annealing on the physical properties of the macroscopic graphene-based papers. Indeed, we demonstrated that the enhancement of the thermal and electrical properties of graphene nanopapers prepared from annealed RGO is strongly influenced by the absence of oxygen functionalities and the morphology of the nanoflakes. Hence, our methodology can be considered as a valid alternative to the classical approach.

## 1. Introduction

Two-dimensional (2D) materials, i.e., one-atom thick layers of van der Waals materials, have gained worldwide attention because of their outstanding properties that arise from their structure and dimensionality [1,2]. Graphene, a single-layer of *sp*^2^ hybridized carbon atoms arranged in a honeycomb lattice, has aroused great interest as one of the most promising 2D materials. The unique physical properties of this carbon allotrope [3,4,5,6,7], in particular its high electron mobility and ballistic conduction [8,9,10], have suggested its use in many technological fields. However, real-world applications such as sensors, electronic devices, thermal management, energy storage conversion and electromagnetic interference (EMI) shielding, to name only a few, often demand the development of flexible, lightweight, paper-like materials with high electrical and thermal conductivity and good corrosion resistance [11,12,13,14,15]. Thus, the fabrication of assembled architectures from graphene building blocks is of fundamental and practical significance to exploit the intrinsic features of individual graphene sheets at a macroscopic level.

Recent advances in the development of flexible graphene paper-like architectures with superior thermal and electrical properties have focused their attention on different assembly strategies [11,14,15,16]. In particular, the flow-directed filtration-induced technique, where graphene suspensions are vacuum filtered, has attracted great interest for the manufacturing of free standing papers [17,18,19,20,21]. Hence, to achieve graphene films with superior properties, individual graphene sheets have to be previously dispersed and stabilized in a liquid medium while the electrostatic repulsive forces and bonding interactions need to be balanced to prevent their re-aggregation [13,17]. A common approach has been the use of graphene oxide (GO) in water suspension as a precursor to obtain GO nanopapers with superior mechanical properties [18,22]. The large interactions at the surface between nanosheets, the wrinkled structure morphology and the crosslinking of the functional groups are responsible for both the high Young’s modulus and mechanical strength [18,20,23,24]. Despite the oxygen-bearing groups facilitate the stabilization of GO in water or polar solvents, they disrupt the *sp*^2^ hybridization of graphene layers, deteriorating the thermal and electrical conductivities of the nanoflakes and, in turn, reducing the performance of the as-prepared GO papers.

In order to restore the π-conjugated system of these materials, two different approaches have been reported. The first strategy is based on the reduction of the as-prepared GO films by chemical methods (using hydrazine hydrate or hydriodic acid), thermal processes or a combination of both. As a result of these processes, the conductivities are partially recovered due to the gradual removal of the oxygen species and water moisture between the nanosheets, packing them more tightly [13,19,25,26]. Nevertheless, the mechanical properties and the structure integrity of the as-prepared GO or reduced graphene oxide (RGO) films are seriously deteriorated after the thermal treatments, even at relatively low temperatures (~200 °C) [25]. The second approach consists in a previous chemical reduction of GO sheets using hydrazine hydrate to produce reduced graphene oxide flakes, followed by sonication in organic solvents and vacuum filtration to produce RGO films [19,20,24,25,27,28,29,30]. In this case, the formation of RGO sheets implies the removal of many functional groups and thus, in order to achieve good dispersions, it is necessary to use organic solvents with surface tension similar to that of graphene such as dimethylformamide (DMF) or *N*-methylpyrrolidone (NMP), the addition of surfactants to the aqueous medium or the formation of graphene colloids. Despite the interactions between oxygen-bearing groups are limited, the vacuum filtration-induced directional-flow allows the assembly of RGO sheets into well-ordered macroscopic structures with remarkable mechanical and electrical properties [19,20,29,31].

We have recently reported that the thermal properties of RGO flakes after thermal annealing at 1700 °C are increased due to the reduction of oxygen-functional groups and the ordering of the graphene structure [32]. This has encouraged us to manufacture conductive graphene nanopapers using an alternative strategy: direct vacuum filtration of both RGO flakes or thermally annealed RGO flakes (RGO_1700) suspensions. In this work, we present the successful fabrication of lightweight nanopapers from both conventional RGO and less defective thermally annealed RGO flakes as well as the assessment of their thermal and electrical properties.

## 2. Materials and Methods

### 2.1. Fabrication of Graphene Nanopapers

RGO and RGO_1700 flakes were prepared accordingly with the previously reported method [32], consisting of highly reduced nanoflakes having lateral size ranging between 0.5 and 2 µm and thickness between 4 and 15 nm (full characterization previously reported in [32]). RGO and RGO_1700 were suspended in DMF at concentrations of 0.15 mg·mL^−^^1^ and the solutions were sonicated in pulsed mode (15 s on and 15 s off) for 15 min with power set at 30% of the full output power (750 W), to avoid significant temperature increase during the treatment, by using an ultrasonication probe (Sonics Vibracell VCX-750, Sonics & Materials Inc., Newtown, CT, USA) with a 13 mm diameter Ti-alloy tip. The RGO and RGO_1700 suspensions were subjected to vacuum filtration using a Nylon Supported membrane (0.45 μm nominal pore size, diameter 47 mm, Whatman, Buckinghamshire, UK). After filtration, the as-obtained papers were peeled off from the membranes and dried at 65 °C under vacuum for 2 h to completely remove the solvent. Finally, RGO and RGO_1700 nanopapers were mechanically pressed in a laboratory hydraulic press (Specac Atlas 15T, Orpington, UK) under a uniaxial compressive load of 5 kN for 10 min at 25 °C.

### 2.2. Characterization

The morphology of the graphene papers was characterized by a high resolution Field Emission Scanning Electron Microscope (FESEM, Zeiss Merlin 4248, Oberkochen, Germany). The thermal diffusivity (*α*) was measured using the xenon light flash technique (LFT) (Netzsch LFA 467 *Hyperflash,* Selb, Germany). The method is compliant with the international standard methods ASTM E-1461, ASTM E-2585, DIN 30905 and DIN EN-821. The samples were cut in disks of diameter 23 mm and the measurements were carried out in a special in-plane sample holder, in which the sample is heated in the central region and the temperature rise is measured on the outer ring of the sample. Measurements were carried out twice using different nanopapers for both RGO and RGO_1700. Each of the samples was measured five times: deviation between diffusivity values was typically found within ±5%. The in-plane thermal conductivity of the film, *k*, was then calculated from the equation *k* = *ρ* × *α* × *C*_p_, where *ρ* is the density of the graphene nanopaper and *C*_p_ is the specific heat capacity of graphite (*C*_p_ = 0.71 J (g·K) ^−^^1^) [33]. The nanopaper density (*ρ*) of the samples was calculated according to the formula *ρ* = *m*/*V*, where *m* is the mass of the nanopaper, weighed using a microbalance (sensitivity: <0.1 µg) and *V* is calculated from a well-defined disk film using the average thicknesses measured as described in [25]. Based on experimental error propagation and sample inhomogeneity (e.g., variations in nanopapers thickness), deviations on calculated conductivity values are estimated within ±10%.

In order to compare the thermal conductivity obtained on nanopapers with different density, owing to different porosity, the conductivity of dense nanoflakes networks were obtained by applying the well-known Maxwell’s effective medium approach [34]. Therefore, the effective conductivity of the network of nanoflakes, *k*_n_, is calculated from Equation (1), where *k* is the thermal conductivity of the nanopaper, *k*_air_ is the thermal conductivity of air and *φ* is the porosity of the nanopapers, i.e., the volume fraction of air, calculated as *φ* = 1 − (*ρ*/*ρ*_g_), where *ρ*_g_ = 2.2 g·cm^−3^ is the density of graphite [35].

(1) $$k=k_{n}\frac{k_{\mathrm{air}}+2k_{n}+2\varphi \left(k_{\mathrm{air}}-k_{n}\right)}{k_{\mathrm{air}}+2k_{n}-\varphi \left(k_{\mathrm{air}}-k_{n}\right)},$$

The electrical conductivity was measured at room temperature by the standard four-point probe method. In order to have a regular geometry, the nanopapers were cut into rectangular stripes with nominal width of 4 mm. The electrical contacts were then fabricated by means of a small drop of conductive silver paste and connected to an Agilent 34420A Nanovoltmeter (Loveland, CO, USA). The conductivity was calculated by the formula $\sigma =\frac{d}{RS\left(1-\varphi \right)}$ where *R* is the measured resistance, *d* the distance between the voltage contacts and *S* = *t* × *w* where *t* and *w* are the thickness and width of the sample, respectively. Conductivity calculation include *φ* to compensate for different densities of the nanopapers, accordingly with the method applied for the thermal conductivity calculation. Uncertainty was evaluated by the propagation of experimental errors on the several measurements used in the calculation and sample inhomogeneity. The most important contributions to the uncertainty of the calculated conductivity values are represented by the deviations in the distance between the voltage electrodes Δ*d*, in the range of ±0.3 mm, due to the finite width of the silver paste spots and the thickness of the nanopapers, Δ*t*, in the range of ±2 µm. A conservative error band was thus estimated in the range of ±15–20%, depending on the specimen quality.

## 3. Results and Discussion

**Figure 1:**
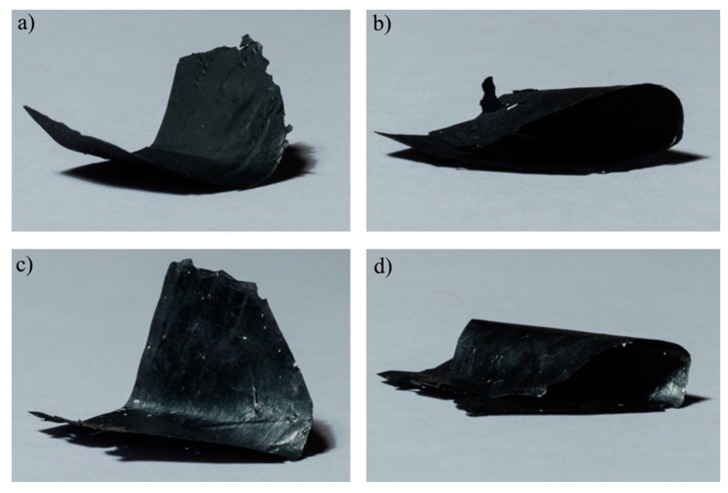
Photographs of free-standing nanopapers (diameter 40 mm): (**a**) RGO nanopaper; (**b**) RGO nanopaper bent 180°; (**c**) RGO_1700 nanopaper; (**d**) RGO_1700 nanopaper bent 180°.

RGO and RGO_1700 were synthesized and characterized as described in our previous work [32]. RGO and RGO_1700 nanopapers were prepared by sonication of the nanoflakes in DMF for 15 min followed by vacuum filtration through a polyamide membrane (see the Materials and Methods Section for details). The resultant nanopapers were peeled off from the membrane, dried under vacuum and mechanically pressed. The fabrication of both graphene-based papers was carried out under the same temperature conditions, concentration of starting material in solution and applied force for compacting the films. The as-obtained free-standing nanopapers are in both cases highly flexible (Figure 1).

**Figure 2:**
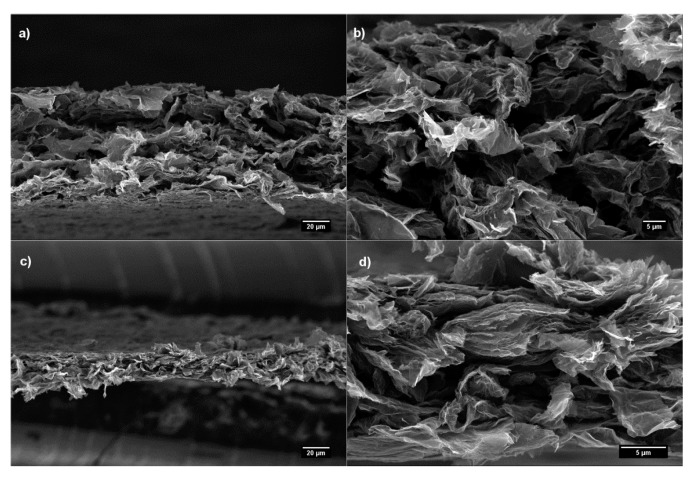
Cross-sectional FESEM images of (**a**) RGO nanopaper and (**c**) RGO_1700 nanopaper and corresponding higher magnifications (**b**,**d**), respectively.

The cross-sectional FESEM images of the free-standing films (Figure 2) reveal a porous-like structure with micrometric cavities between the nanoflakes. The formation of irregularly stacked nanoflakes assemblies by vacuum filtration follows a semi-ordered accumulation mechanism [36,37]. According to this process, the randomly oriented graphene flakes within a suspension form a loosely aggregated structure during the first stages of the filtration due to the weak adhesion between adjacent nanoflakes. The compressive forces generated as the solvent is removed, perpendicular to the direction of the solvent flow, produce a significant degree of order in the structure, even if this process does not completely align the resultant papers, as observed in the FESEM images. Furthermore, the crumpled structure of the initial RGO nanoflakes limits the packing of flakes in the nanopaper and cause the formation of microsized air cavities, leading to an average thickness of 34 ± 2 µm and resulting in a measured density of 0.4 g·cm^−3^. The RGO_1700 nanopaper (Figure 2c,d) shows a more tightly packed and homogeneous structure compared to the RGO nanopaper, with an average thickness of 20 ± 2 µm (Figure 2a,b). This difference may be attributed to both physical and chemical reasons. On the one hand, the thermal annealing is known to increase order within the stack of graphene layers in the thickness of a RGO nanoflake: indeed, annealing at high temperature was previously reported to increase correlation lengths both in-plane and perpendicular to the plane of the nanoflakes [32]. Despite the overall morphology of the nanoflakes remains rather crumpled [32], the increase in correlation length may result in a higher planarity at least in some regions of the nanoflakes, thus affecting the efficiency of nanoflakes stacking. On the other hand, the annealing treatment of the RGO flakes at 1700 °C reduces the number of oxidized groups on the nanoplatelets. The lack of significant oxidation reduces the steric hindrance arising from the residual functional groups, and enhances the possibility to build π-π interactions over extended areas, thus contributing in more efficient packing of flakes into the nanopaper [38], finally leading to a density of 1.0 g·cm^−3^ for RGO_1700 nanopapers.

Graphene-based nanopapers are microscopically heterogeneous materials which can be considered as graphene-air composites. Hence, the thermal and electrical conductivities of graphene-based nanopapers are dominated by their intrinsic structure which strongly depends not only on the intrinsic conductivity of the individual nanoflakes, but also on the size of the nanoplatelets, their orientation in the films, the porosity and the contact resistance [39].

In-plane thermal diffusivity for both RGO and RGO_1700 nanopapers was measured by xenon flash method. The RGO nanopaper showed a diffusivity value of 43 ± 2 mm^2^·s^−1^ at 25 °C. A slight reduction of diffusivity was measured when increasing the temperature to 50 °C (40 ± 2 mm^2^·s^−1^) and 100 °C (35 ± 2 mm^2^·s^−1^), which is expected owing to phonon Umklapp-scattering [6]. A diffusivity value of 198 ± 10 mm^2^·s^−1^ for the RGO_1700 nanopaper was obtained at 25 °C, clearly evidencing a better performance of the nanopaper obtained with annealed RGO nanoflakes. However, due to the significant differences in density and thickness between nanopapers prepared with pristine and annealed nanoflakes, such figures represent the performance in heat spreading on the different nanopapers (i.e., the manufacts) and not the intrinsic property of the dense network made of RGO/RGO_1700 nanoflakes. While the in-plane thermal conductivity of the nanopaper can simply be calculated from in-plane diffusivity measurements multiplied by its density and heat capacity, a comparison between the intrinsic thermal conductivity of the dense networks made of RGO or RGO_1700 should be made by decoupling from the nanopaper density. Conductivity values of the nanoflake networks were therefore obtained by applying the Maxwell’s effective medium approach, as detailed in the experimental section.

The in plane thermal conductivity values of the dense nanoflakes networks were calculated according to this model giving, within ±10% error, 95 W·m^−1^·K^−1^ for the RGO nanopaper and 390 W m^−1^·K^−1^ for the RGO_1700 one at 25 °C, thus evidencing a dramatic increase of thermal transfer performance of the carbon nanoflakes network upon high-temperature annealing of the RGO nanoflakes. The enhancement of the thermal dissipation of the individual RGO flakes after thermal annealing, as a consequence of the removal of oxygen-functional groups with a simultaneous restoration of the *sp*^2^ hybridization of the C atoms, has already been proved by Scanning Thermal Microscopy (SThM) [32], even though a quantitative determination of the thermal conductivity from SThM measurements remains extremely challenging [40]. Here we demonstrate that the improvement of the thermal transfer efficiency for the annealed RGO flakes is directly correlated to a dramatic increase by a magnitude factor of four at the macroscale.

Beside the effect of annealing, it is worth noting that the thermal conductivity of the nanopaper prepared in this work by filtration of RGO is higher to that reported by Renteria et al. for their dense rGO nanopaper prepared by deposition of GO [11], thermally reduced at 1000 °C, thus confirming the preparation method proposed in this work as a valid alternative to the conventional method. In fact, annealing rGO powder could routinely be carried out after conventional thermal reduction, and the obtained powders may be exploited for the preparation of nanopapers of large size, including by continuous processes, thus overcoming the limitation in terms of maximum size for the post treatment of the nanopapers in the traditional method. The thermal conductivity of the present nanopaper is also significantly higher than the previously reported graphene laminate films on polymer substrate [41], despite a proper comparison between these two materials should take into account the obvious difference in the type, thickness and defectiveness of the nanoflakes, the presence of surfactants in commercial ink as well as the role of the polymer substrate.

We further studied the electrical conductivity of the RGO and RGO_1700 nanopapers using the four-point probe method. Electrical conductivities measured were normalized on RGO flakes volume fraction, accordingly with the method applied in thermal conductivity. The electrical conductivity increases from (5.8 ± 0.7) × 10^3^ S·m^−1^ for the RGO flakes, to (2.1 ± 0.4) × 10^4^ S·m^−1^ for the annealed ones. These results are in accordance with the improvements observed in the thermal properties. It is known that defects and incomplete reduction hinder the electrical performance of RGO, in terms of both reduced electrical conductivity and charge carrier mobility and the thermal treatment favours the increase of *sp*^2^ domains and thus the graphitization of the nanoflakes [11,42]. In the literature, GO nanopapers prepared in a similar way and treated at 800 and 700 °C showed electrical conductivities of 5.8 × 10^3^ and 5.0 × 10^3^ S·m^−1^, respectively [27,43], respectively, consistent with the values observed in this work for RGO papers, confirming the effectiveness of our preparation approach for nanopapers, as observed for the thermal properties.

## 4. Conclusions

In summary, our procedure to prepare graphene-based nanopapers by flow-directed filtration-induced technique using mild conditions and without post-treatments is effective to produce highly porous and flexible macroscopic all-graphene based structures. The structure of the resultant graphene nanopapers is strongly influenced by the defectiveness of the starting RGO flakes. The thermal and electrical properties were improved in nanopapers prepared with thermally treated RGO flakes, because the reduction of functional groups not only restores the *sp*^2^ carbon structure but also generates the formation of higher density structures. The combination of the thermal and electrical properties accompanied by the low density of these macroscopic graphene-based materials makes these assembled architectures potential candidates in thermal management and electronic applications.
